# Analysis of “true extrauterine growth retardation” and related factors in very preterm infants—A multicenter prospective study in China

**DOI:** 10.3389/fped.2022.876310

**Published:** 2022-09-15

**Authors:** Wei Shen, Fan Wu, Jian Mao, Ling Liu, Yan-Mei Chang, Rong Zhang, Zhi Zheng, Xiu-Zhen Ye, Yin-Ping Qiu, Li Ma, Rui Cheng, Hui Wu, Dong-Mei Chen, Ling Chen, Ping Xu, Hua Mei, San-Nan Wang, Fa-Lin Xu, Rong Ju, Chao Chen, Xiao-Mei Tong, Xin-Zhu Lin

**Affiliations:** ^1^Department of Neonatology, Children’s Hospital of Fudan University, Shanghai, China; ^2^Department of Neonatology, Women and Children’s Hospital, School of Medicine, Xiamen University, Xiamen, Fujian, China; ^3^Department of Neonatology, The Third Affiliated Hospital of Guangzhou Medical University, Guangzhou, Guangdong, China; ^4^Department of Pediatrics, Shengjing Hospital of China Medical University, Shenyang, China; ^5^Department of Neonatology, Guiyang Maternal and Child Health Hospital (Guiyang Children’s Hospital), Guiyang, Guizhou, China; ^6^Department of Pediatrics, Peking University Third Hospital, Beijing, China; ^7^Department of Neonatology, Guangdong Province Maternal and Children’s Hospital, Guangzhou, Guangdong, China; ^8^Department of Neonatology, General Hospital of Ningxia Medical University, Yinchuan, Ningxia, China; ^9^Department of Neonatology, Children’s Hospital of Hebei Province, Shijiazhuang, Hebei, China; ^10^Department of Neonatology, Children’s Hospital of Nanjing Medical University, Nanjing, Jiangsu, China; ^11^Department of Neonatology, The First Hospital of Jilin University, Changchun, Jilin, China; ^12^Department of Neonatology, Quanzhou Maternity and Children’s Hospital, Quanzhou, Fujian, China; ^13^Department of Pediatrics, Tongji Hospital, Tongji Medical College, Huazhong University of Science and Technology, Wuhan, Hubei, China; ^14^Department of Neonatology, Liaocheng People’s Hospital, Liaocheng, Shandong, China; ^15^Department of Neonatology, The Affiliated Hospital of Inner Mongolia Medical University, Hohhot, Inner Mongolia, China; ^16^Department of Neonatology, Suzhou Municipal Hospital, Suzhou, Jiangsu, China; ^17^Department of Neonatology, The Third Affiliated Hospital of Zhengzhou University, Zhengzhou, Henan, China; ^18^Department of Neonatology, Chengdu Women’s and Children’s Central Hospital, School of Medicine, University of Electronic Science and Technology of China, Chengdu, Sichuan, China; ^19^Xiamen Key Laboratory of Perinatal-Neonatal Infection, Xiamen, Fujian, China

**Keywords:** very preterm infants, extrauterine growth retardation, “true EUGR”, nutrition, risk factor

## Abstract

**Objective:**

To investigate the incidence and related factors of extrauterine growth retardation (EUGR) and “true EUGR” in very preterm infants (VPI) from different regions of China.

**Materials and methods:**

Clinical data of VPI were prospectively collected from 28 hospitals in seven different regions of China from September 2019 to December 2020. The infants were divided into a small for gestational age (SGA) group or non-SGA group at birth, with non-SGA infants at 36 weeks of gestation or at discharge being further divided into a EUGR group or a non-EUGR group. Infants in the EUGR and non-SGA group were defined as “true EUGR.” The general information of VPI, such as maternal complications during pregnancy, use of enteral nutrition and parenteral nutrition, and complications during hospitalization were compared between the groups.

**Results:**

Among the 2,514 VPI included in this study, 47.3, 41.5, and 33.3% of VPI were below the 10th percentile, and 22.6, 22.4, and 16.0% of VPI were below the 3rd percentile for weight, height, and head circumference at 36 weeks of gestation or at discharge, respectively, by the percentile on the 2013 Fenton curve. The incidences of EUGR and “true EUGR” evaluated by weight were 47.3 and 44.5%, respectively. Univariate analysis showed that there were statistically significant differences in the aspects of perinatal and nutritional characteristics, treatment, and complications between the groups. Multivariate analysis showed that in non-SGA infants, the cumulative caloric intake during the first week was a protective factor for “true EUGR,” while days to reach total enteral nutrition, late initiation of human milk fortifier, and moderate to severe bronchopulmonary dysplasia were independent risk factors for “true EUGR.”

**Conclusion:**

More attention should be paid to the nutritional management of VPI to prevent “true EUGR.” Cumulative caloric intake should be ensured and increased during the first week, total enteral nutrition should be achieved as early as possible, human milk fortifier should be added early, and moderate to severe bronchopulmonary dysplasia should be prevented. These strategies are very important for reducing the incidence of “true EUGR” in VPI.

## Introduction

The prevalence of very preterm infants (VPI) is increasing all over the world (1). Despite progress in the survival of VPI (preterm infants born at less than 32 weeks’ gestation), they remain at a significantly higher risk of developing health and developmental problems when compared to full-term infants. Previous research indicates that VPI have a high prevalence of extrauterine growth retardation (EUGR), which has attracted the attention of neonatologists as these infants experience more long-term medical problems after discharge. Extrauterine growth retardation is a continuation of small for GA (SGA), and intrauterine developmental delay will lead to a delay in postnatal growth and development (2). The growth indicators of SGA infants do not reach the 10th percentile as compared to infants of the same corrected GA at discharge, and it takes longer to complete the catch-up growth. This has been related to the intrauterine growth retardation, inadequate nutritional reserve, relative immaturity of organs, and multiple complications after birth (3). Studies have shown that EUGR not only affects the physical development and short-term complications of premature infants, but it also affects their long-term health, especially neurocognitive function, and increases the risk of cardiovascular disease and chronic metabolic syndrome (4–6). Hence, improving the level of perinatal medical technology, strengthening perinatal health care, ensuring adequate nutritional intake before and during pregnancy, and reducing the occurrence of SGA are crucial for reducing the occurrence of EUGR. A promising area for improving the prognosis of VPI is postnatal nutrition and growth (7, 8). Thus, understanding and optimizing the growth of VPI during the neonatal intensive care unit stay remains an important topic that has short- and long-term health implications for the infants.

Figueras-Aloy et al. (2) have suggested a new term, “true EUGR”, which refers to cases of EUGR without any evidence of fetal growth impairment (SGA at birth). The term “true EUGR” was used to differentiate EUGR related to postnatal growth failure among non-SGA infants from pre-programed EUGR related to inadequate antenatal nutritional status among SGA infants (9). Postnatal growth restriction in SGA infants is likely not of postnatal origin, but a continuation of the process that has previously affected fetal growth. Therefore, Figueras-Aloy et al. (2) have recommended that in studies of prevention or trials testing treatments or nutritional regimens for EUGR, the preterm subsample with SGA should be clearly differentiated from non-SGA.

In recent years, increasing attention has been paid to the prevention and treatment of nutritional deficiency and EUGR in preterm infants in China (10, 11), but there are very few national multicenter studies on the incidence and risk factors of EUGR, especially “true EUGR” in VPI. Thus, the aim of this study was to prospectively investigate the clinical data of VPI to understand the incidence of “true EUGR” at discharge in China and analyze the related influencing factors, and to provide a scientific basis for optimizing the hospital nutrition support strategy and effective prevention and treatment of “true EUGR” in VPI.

## Subjects and methods

### Research subjects

#### Recruitment of very preterm infants

Participants for this study were recruited from 28 tertiary hospitals located in seven different regions of China (northeast, north, east, central, south, northwest, and southwest) and the hospitals included 13 general hospitals, 11 children’s hospitals, and four maternal and childcare hospitals.

#### Categorization of very preterm infants

Participants were divided into the SGA group and non-SGA group at birth, and the non-SGA infants at the corrected GA of 36 weeks (36 weeks PCA) or at discharge (when before 36 weeks PCA) were further divided into the EUGR group and non-EUGR group, according to whether their body weight, length, and head circumference (HC) were below the 10th percentile on the Fenton curve.

#### Inclusion criteria, exclusion criteria, and discharge criteria

Inclusion criteria were as follows: (1) GA < 32 w; (2) Length of hospital stay > 2 weeks; (3) Hospital admission within 24 h after birth.

Exclusion criteria were as follows: (1) Congenital malformation or inherited metabolic disease; (2) Hospital stay < 2 weeks; (3) Death during hospitalization, interruption of treatment, or automatic discharge.

Discharge criteria were as follows: (1) The primary disease was cured, and the vital signs were stable [infants with bronchopulmonary dysplasia (BPD) were allowed to be discharged with oxygen]; (2) milk volume had reached total enteral feeding; (3) Weight was more than 1,800–2,000 g; (4) Corrected GA was ≥ 36 weeks.

#### Ethics approval and clinical trial registration

This study was conducted by the Nutrition Professional Committee of Neonatologists Branch of Chinese Medical Doctor Association, and it was registered in the Chinese Clinical Trial Registry (Registration number: ChiCTR1900023418). The research protocol was approved by the Ethics Committee of Women and Children’s Hospital affiliated to Xiamen University/Xiamen Maternal and Child Health Hospital (Batch number kY-2019-016).

### Research methods

#### Study design

This was a multicenter prospective study, and the study period was from September 2019 to December 2020.

#### Data collection

Using a unified questionnaire, data on the maternal complications during pregnancy, general clinical data of preterm infants, nutritional status during hospitalization, complications, and major treatments were collected. Perinatal data included GA at birth, birth weight (BW), sex, delivery mode, single or multiple births, 1-min Apgar score, and full course of prenatal glucocorticoid use. Maternal complications during pregnancy included gestational hypertension, gestational diabetes, thyroid disease, and connective tissue disease. Growth and nutrition related indicators included maximum weight loss, days to regain BW, growth velocity (GV) after regaining BW, start of enteral nutrition (EN), age at reaching total EN, duration of parenteral nutrition (PN), cumulative fasting days, calories accumulated during the first week of hospitalization, days to reach the target total calorie intake and oral calorie intake, accumulative doses of amino acid and fat emulsions during the first week of hospitalization and throughout hospitalization, breast milk volume after addition of human milk fortifier (HMF), and days needed for full fortification. The main treatments included duration of invasive and non-invasive mechanical ventilation, duration of oxygen use, cumulative duration of antibiotic use, and total length of hospital stay. Primary complications during hospitalization included neonatal respiratory distress syndrome (RDS), moderate to severe BPD, early-onset sepsis (EOS), late-onset sepsis (LOS), necrotizing enterocolitis (NEC) ≥ stage 2, intraventricular hemorrhage (IVH) grade III-IV, periventricular leukomalacia (PVL), hemodynamically significant patent ductus arteriosus (hsPDA), retinopathy of prematurity (ROP), feeding intolerance (FI), metabolic bone disease of prematurity (MBDP), and PN associated cholestasis (PNAC).

#### Data management and quality control

The data entry personnel of each unit were uniformly trained, and they strictly fulfilled the requirements of the research program. The EpiData database was established in this study, data of the case report form were recorded in double pairs, all participating units collected and uploaded the nutrition data of preterm infants in time, and the database was locked after verification. The team leader maintained close contact with all participating units at any time point, checked the case records, and solved the possible problems in time.

#### Definitions, diagnostic criteria, and calculation methods of related diseases

(1) The term “SGA” referred to BW below the 10th percentile of the average BW of infants of the same sex and GA; the term ‘appropriate for gestational age’ was defined as BW within the 10th to 90th percentile of the BW of infants of the same sex and age; and the term “large for gestational age” referred to BW above the 90th percentile of the BW for infants of the same sex and age. (2) Evaluation criteria for EUGR: With reference to Fenton 2013 (12), EUGR was defined when weight, length, and HC were below the 10th percentile on the growth curve at the corrected GA of 36 weeks or at discharge, and severe EUGR was defined when the growth parameters were below the 3rd percentile on the growth curve. Infants in the EUGR and non-SGA group were defined as “true EUGR.” “True-EUGR,” which refers to cases of EUGR without evidence of fetal growth impairment (SGA at birth). (3) Moderate to severe BPD was defined as requirement of oxygen therapy, positive pressure ventilation, or mechanical ventilation at the corrected GA of 36 weeks or at discharge. (4) Hemodynamically significant patent ductus arteriosus (hsPDA) was defined as patent ductus arteriosus (PDA) catheter diameter > 1.5 mm, left atrial diameter/aortic diameter ≥ 1.4 mm, or left ventricular end-diastolic diameter/aortic diameter ≥ 2.1 mm accompanied by one of the following clinical manifestations: heart murmur, tachycardia (sustained ≥ 160 beats/min), increased respiration, increased pulse pressure (>25 mmHg), hypotension, flushing, or cardiac dilation. (5) Interventional ROP was defined as ROP requiring intravitreal drug injection, laser therapy, or surgery. (6) Days to reach total EN was defined as time required for oral milk feeding to reach 150 ml/kg/day; days to reach the target total calorie intake and oral calorie intake was defined as time required for the calorie intake to reach 110 kcal/kg/day. (7) Growth velocity after regaining BW was calculated as follows: GV = [1000 × ln (Wn/W1)]/(Dn–D1) (13), Wn, discharged weight; W1, BW; Dn, length of hospital stay; D1, days to regain BW. (8) The diagnoses of RDS, EOS, LOS, NEC ≥ stage 2, IVH grade III-IV, PVL, FI, MBDP, PNAC, and anemia were established by referring to Practical Neonatology (5th edition) (14).

#### Sample size calculation

According to the literature, the incidence of EUGR was about 60% and the allowable error (δ) was 2%. If we considered α = 0.05, the required sample size was 2,305 and the required total sample size was 2,400. The average sample size for each collaborating unit was expected to be 90.

### Statistical methods

All data were analyzed with SPSS 23.0 for Windows (SPSS Inc., Chicago, IL, United States). Chi-square test was used to assess the association between categorical variables unless the cell frequency was ≤ 5, in which case the Fisher’s exact test was used. Distribution of continuous variables was assessed using the Shapiro–Wilk test. Normally distributed continuous variables were analyzed using independent sample *t*-test, while Mann–Whitney U test was used for non-normal distributions. Univariate analysis was performed for the factors that may affect the clinical outcome of infants. To determine the effect of the prenatal and postnatal variables on the outcome of true EUGR, binomial logistic regression was used to arrive at odds ratios (OR) and 95% confidence intervals (CI). In the multivariate model, true EUGR (yes or no) was considered as the dependent variable, and nutritional factors and complications related variables were analyzed as independent variables. The Hosmer–Lemeshow test was conducted to determine the model’s goodness of fit. All statistical tests were two-tailed, and *P*-values ≤ 0.05 were considered statistically significant.

## Results

### The incidence of extrauterine growth retardation and “true extrauterine growth retardation” in very preterm infants at discharge

**FIGURE 1:**
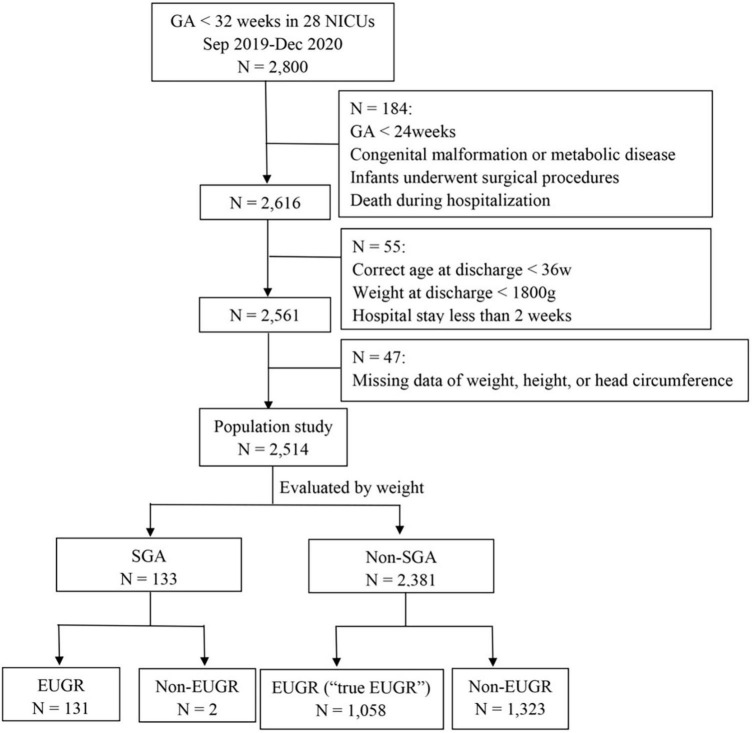
The flow chart of the included infants. GA, gestational age; NICU, neonatal intensive care unit; SGA, small for gestational age; EUGR, extrauterine growth retardation.

A total of 2,800 preterm infants with GA < 32 weeks were admitted during the study period. A total of 184 preterm infants, aged < 24 weeks at birth, died during hospitalization, or had severe congenital developmental abnormalities, inherited metabolic diseases, chromosomal abnormalities, or underwent surgical procedures, were excluded. A total of 55 infants were excluded from the study as the corrected age was less than 36 weeks, their weight was less than 1,800 g at discharge, or the hospital stay was less than 2 weeks. A total of 47 infants were excluded because of missing data on weight, height, or HC. Finally, 2,514 VPI were enrolled in the study. The flow chart of the included infants is shown in Figure 1.

**FIGURE 2:**
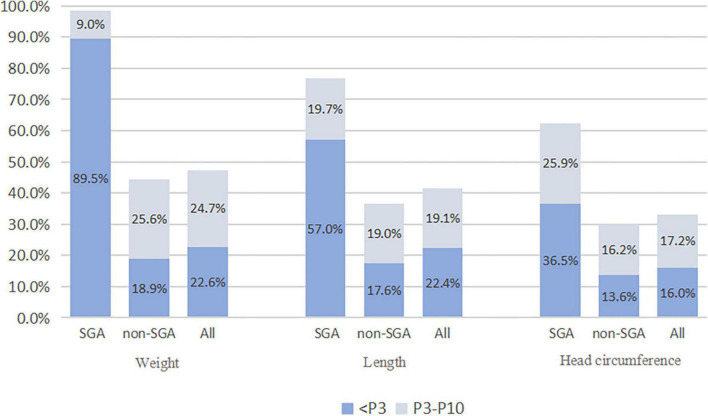
The incidence of EUGR in SGA and non-SGA infants. SGA, small for gestational age; EUGR, extrauterine growth retardation.

The infants were divided into the SGA and non-SGA groups at birth, and non-SGA infants at 36 weeks of gestation or at discharge were further divided into the EUGR group and non-EUGR group. According to weight, the incidence of EUGR was 98.5, 44.5, and 47.3% in SGA infants, non-SGA infants, and all 2,514 infants, respectively; and the incidences of EUGR and “true EUGR” evaluated by weight were 47.3 and 44.5%, respectively. According to body length, the incidence of EUGR was 76.7, 36.6, and 41.5% in SGA infants, non-SGA infants, and all infants. When evaluated by HC, the incidence of EUGR was 62.4, 29.8, and 33.2% in SGA infants, non-SGA infants, and all infants, respectively. The incidence of EUGR in SGA and non-SGA infants, and the specific proportions of infants who had values less than P3 and P10 at discharge in each group are shown in Figure 2.

**FIGURE 3:**
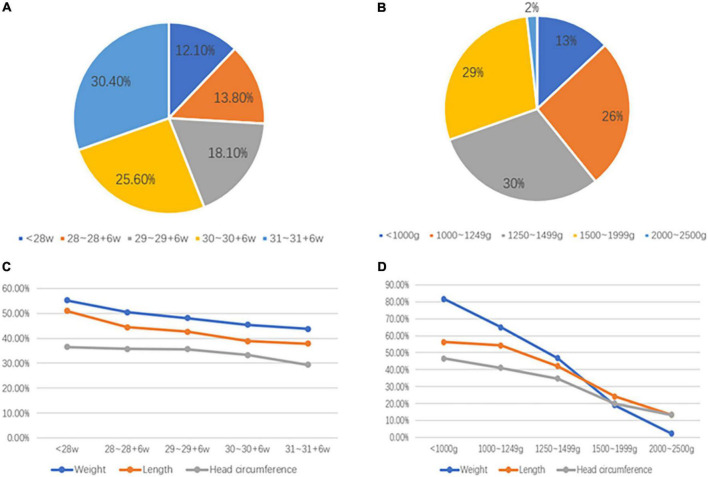
The distribution of gestational age and birth weight and incidence of EUGR. (**A**) The distribution of gestational age; (**B**) The distribution of birth weight; (**C**) Incidence of EUGR stratified by gestational age, evaluated by weight, length, and head circumference; (**D**) Incidence of EUGR stratified by birth weight, evaluated by weight, length, and head circumference. EUGR, extrauterine growth retardation.

Figures 3A,B show the distribution of GA and BW in these 2,514 cases, respectively. Figure 3C shows the incidence of EUGR stratified by GA, as evaluated by weight, length, and HC. The difference in the incidence of EUGR was statistically significant between the age groups (*P* < 0.05, data not shown). Preterm infants with GA < 28 weeks had the highest incidence of EUGR (55.3, 51.0, and 36.5%, for weight, length, and HC, respectively), and those with GA 31-31 + 6 weeks had the lowest incidence of EUGR (43.8, 37.9, and 29.4%, for weight, length, and HC, respectively). Figure 3D shows the incidence of EUGR stratified by BW, as evaluated by weight, length, and HC. The difference in the incidence of EUGR was also statistically significant between the BW groups (*P* < 0.05, data not shown). Preterm infants with BW < 1,000 g had the highest incidence of EUGR (81.7, 56.3, and 46.5%, for weight, length, and HC, respectively), and those with BW 2,000–2,500 g had the lowest incidence of EUGR (2.2, 13.3, and 13.3%, for weight, length, and HC, respectively).

### Univariate analysis of the influencing factors of “true extrauterine growth retardation” evaluated by weight

Comparisons of obstetrical and neonatal characteristics of VPI are shown in Table 1. The incidences of cesarean delivery, 1 min Apgar score ≤ 7, and gestational hypertension were higher in the SGA group than in the non-SGA group, and the respective incidences were higher in the EUGR group than in the non-EUGR group (*P* < 0.05). The incidence of gestational diabetes was higher in the non-SGA group and non-EUGR group when compared with the SGA group and EUGR group, respectively (*P* < 0.05). There was a tendency that the incidence of full course of prenatal steroids was higher in the SGA group (*P* = 0.072), and at discharge, this incidence was higher in the non-EUGR group (*P* < 0.05).

**TABLE 1:** Comparisons of obstetrical and neonatal characteristics of very preterm infants (VPI) between the groups.

	At birth (*n* = 2,514)		X	*P*	Non-SGA group (*n* = 2,381)		X	*P*
	SGA (*n* = 133)	Non-SGA (*n* = 2,381)			EUGR (true EUGR) (*n* = 1,058)	Non-EUGR (*n* = 1,323)		
Male [*n* (%)]	76 (57.1)	1,302 (54.7)	0.358	0.836	567 (53.6)	735 (55.6)	2.074	0.354
Cesarean delivery [*n* (%)]	123 (92.5)	1,413 (59.4)	58.115	0.000	684 (64.7)	729 (55.1)	22.018	0.000
Multiple pregnancies [*n* (%)]	43 (32.3)	777 (32.7)	0.006	0.937	366 (34.6)	411 (31.1)	3.236	0.072
Apgar ≤ 7 (1 min) [*n* (%)]	65 (48.9)	859 (36.1)	8.871	0.003	452 (42.7)	407 (30.8)	36.456	0.000
Prenatal steroids [*n* (%)]	112 (84.2)	1,847 (77.6)	3.227	0.072	791 (74.8)	1,056 (79.8)	8.634	0.003
Gestational hypertension [*n* (%)]	82 (61.7)	435 (18.3)	145.137	0.000	281 (26.6)	154 (11.6)	87.634	0.000
Gestational diabetes [*n* (%)]	12 (9.0)	425 (17.8)	6.834	0.009	162 (15.3)	263 (19.9)	8.363	0.004
Maternal thyroid disease [n (%)]	6 (4.5)	126 (5.3)	0.154	0.694	60 (5.7)	66 (5.0)	0.546	0.460
Maternal connective tissue disease [*n* (%)]	4 (3.0)	32 (1.3)	2.470	0.116	15 (1.4)	17 (1.3)	0.078	0.780

SGA, small for gestational age; EUGR, extrauterine growth retardation; true EUGR, infants in the EUGR and non-SGA group.

Table 2 shows comparisons of the growth conditions. Gestational ages at birth and at discharge were higher in the SGA group than in the non-SGA group. Gestational age was higher in the non-EUGR group at birth and in the EUGR group at discharge (all *P* < 0.05). For weight, length, HC, the corresponding Z score, and the change in Z score, all except two indicators were significant between the SGA and non-SGA groups at birth, and between the EUGR and non-EUGR groups belonging to the non-SGA group at discharge (*P* < 0.05). Changes in weight Z score and HC at discharge were not significantly different between the SGA and non-SGA groups at birth (*P* = 0.642 and 0.891, respectively). However, they became significant between the EUGR and non-EUGR groups belonging to the non-SGA group at discharge (*P* < 0.05).

**TABLE 2:** Comparisons of growth between the groups.

	At birth (*n* = 2,514)		X	*P*	Non-SGA group (*n* = 2,381)		X	*P*
	SGA (*n* = 133)	Non-SGA (*n* = 2,381)			EUGR (true EUGR) (*n* = 1,058)	Non-EUGR (*n* = 1,323)		
**Gestational age M (P25, P75)**								
At birth (w)	30.86 (29.86, 31.57)	30.14 (28.86, 31.14)	–5.167	0.000	29.93 (28.57, 31.00)	30.28 (29.00, 31.14)	–4.795	0.000
At discharge (w)	38.43 (37.50, 39.93)	36.43 (35.43, 37.71)	–11.950	0.000	37.14 (36.43, 38.43)	35.86 (35.00, 36.86)	–21.989	0.000
**Weight M (P25, P75)**								
At birth (g)	938 (780, 1,090)	1,350 (1,160, 1,550)	–15.340	0.000	1,220 (1,050, 1,390)	1,470 (1,280, 1,650)	–20.510	0.000
Z score at birth	–1.51 (–1.68, –1.39)	0.07 (–0.38, 0.51)	–19.436	0.000	–0.30 (–0.74, 0.13)	0.32 (–0.04, 0.74)	–23.229	0.000
At discharge (g)	2,050 (1,970, 2,150)	2,250 (2,050, 2,500)	–8.178	0.000	2,120 (1,999, 2,350)	2,340 (2,150, 2,620)	–16.338	0.000
Z score at discharge	–2.73 (–3.25, –2.31)	–1.14 (–1.69, –0.64)	–16.362	0.000	–1.76 (–2.18, 1.48)	–0.71 (–0.99, –0.33)	–41.777	0.000
Change in Z score (ΔZ)	1.17 (0.80, 1.58)	1.22 (0.82, 1.65)	–0.464	0.642	1.56 (1.18, 2.06)	0.97 (0.62, 1.33)	–23.496	0.000
**Length M (P25, P75)**								
At birth (cm)	35.0 (33.0, 37.0)	39.0 (37.0, 41.0)	–12.425	0.000	38.0 (35.4, 40.0)	40.0 (38.0, 41.0)	–15.590	0.000
Z score at birth	–1.79 (–2.43, –1.24)	0.00 (–0.63, 0.57)	–16.756	0.000	–0.33 (–0.92, 0.29)	0.29 (–0.28, 0.75)	–15.564	0.000
At discharge (cm)	43.5 (42.0, 45.0)	45.0 (43.5, 46.5)	–6.955	0.000	44.5 (43.0, 46.0)	45.0 (44.0, 47.0)	–8.920	0.000
Z score at discharge	–2.58 (–3.44, –1.90)	–1.01 (–1.67, –0.38)	–14.156	0.000	–1.56 (–2.19, –1.02)	–0.59 (–1.1, –0.10)	–25.323	0.000
Change in Z score (ΔZ)	0.81 (0.03, 1.65)	1.04 (0.38, 1.71)	–2.046	0.041	1.30 (0.57, 2.00)	0.85 (0.27, 1.47)	–10.016	0.000
**Head circumference M (P25, P75)**								
At birth (cm)	26.0 (24.1, 27.0)	27.0 (26.0, 29.0)	–8.748	0.000	27.0 (25.5, 28.0)	28.0 (26.5, 29.0)	–12.523	0.000
Z score at birth	–1.36 (–2.12, –0.82)	0.13 (–0.47, 0.77)	–14.863	0.000	–0.15 (–0.82, 0.48)	0.33 (–0.24, 0.95)	–12.738	0.000
At discharge (cm)	31.9 (31.0, 32.5)	31.5 (30.8, 32.5)	–0.137	0.891	31.5 (30.5, 32.4)	32.0 (31.0, 33.0)	–4.786	0.000
Z score at discharge	–1.81 (–2.46, –1.30)	–0.84 (–1.46, –0.22)	–10.808	0.000	–1.30 (–1.89, –0.77)	–0.43 (–0.99, 0.08)	–22.590	0.000
Change in Z score (ΔZ)	0.44 (–0.31, 1.03)	0.95 (0.29, 1.68)	–5.958	0.000	1.17 (0.36, 1.94)	0.83 (0.22, 1.49)	–7.388	0.000
**Other growth indicators M (P25, P75)**								
The maximum weight loss (%)	5.4 (1.5, 8.0)	6.4 (3.7, 9.2)	–3.273	0.001	6.5 (3.7, 9.7)	6.3 (3.7, 9.0)	–1.312	0.190
Days to regain BW (d)	7.5 (5.0,10.8)	9.0 (7.0,12.0)	–4.189	0.000	9.0 (7.0,12.0)	9.0 (7.0,11.0)	–2.852	0.004
Weight gain velocity [g/kg/day]	16.6 (14.6,19.1)	15.0 (12.7,18.2)	–4.472	0.000	14.0 (11.8,16.8)	15.9 (13.6,19.4)	–11.581	0.000

SGA, small for gestational age; EUGR, extrauterine growth retardation; true EUGR, infants in the EUGR and non-SGA group; w, week; g, gram; d, day; BW, birth weight.

Comparisons of nutrition-related characteristics between the groups are shown in Table 3. Compared with the non-SGA group and non-EUGR group, in the SGA group and EUGR group, the duration of PN was longer, the number of cumulative fasting days was higher, the rate of central vein use was higher, and the attainment of total EN, and total calorie and oral calorie intake was delayed (*P* < 0.05). The cumulative dose of amino acid and fat emulsions during the first week (W1) and during hospitalization was higher in the SGA group and EUGR group (*P* < 0.05). When comparing the indicators for the start of EN, cumulative calories during W1, breast milk volume after addition of HMF, and days taken for full fortification, the differences were not significant between the SGA and non-SGA groups at birth, but they became significant between the EUGR and non-EUGR groups belonging to the non-SGA group at discharge (*P* < 0.05).

**TABLE 3:** Comparisons of nutrition-related characteristics between the groups.

	At birth (*n* = 2,514)		X	*P*	Non-SGA group (*n* = 2,381)		X	*P*
	SGA (*n* = 133)	Non-SGA (*n* = 2,381)			EUGR (true EUGR) (*n* = 1,058)	Non-EUGR (*n* = 1,323)		
The start of EN (h) M (P25, P75)	24.0 (9.3, 48.8)	23.0 (8.0, 40.0)	–1.535	0.125	24.0 (12.0, 48.0)	20.0 (6.0, 30.0)	–7.767	0.000
Days to reach total EN (d) M (P25, P75)	30.0 (23.0, 41.0)	25.0 (17.0, 35.0)	–4.762	0.000	30.0 (21.0, 40.0)	21.0 (15.0, 30.0)	–13.694	0.000
Duration of PN (d) M (P25, P75)	27.0 (20.0, 36.0)	20.0 (13.0, 30.0)	–5.616	0.000	24.0 (17.0, 35.0)	18.0 (11.0, 26.0)	–12.977	0.000
Use of the central vein [*n* (%)]	127 (96.2)	1927 (81.2)	19.136	0.000	936 (88.6)	991 (75.2)	69.359	0.000
Cumulative fasting time (d) M (P25, P75)	3.0 (1.0, 7.0)	2.0 (0.8, 5.0)	–3.273	0.001	3.0 (1.0, 6.9)	1.0 (0.4, 3.0)	–12.759	0.000
**Calories M (P25, P75)**								
Cumulative calories during W1 (kcal/Kg)	490.7 (420.9, 556.9)	497.0 (421.0,565.0)	–0.798	0.425	480.0 (410.0, 546.8)	512.6 (433.3,579.4)	–6.701	0.000
Age at reaching the target total calorie intake (110 kcal/kg) (d)	11.0 (8.0, 17.0)	9.0 (7.0, 14.0)	–3.525	0.000	11.0 (7.0, 17.0)	8.0 (6.0, 12.0)	–10.717	0.000
Age at reaching the target oral calorie intake (110 kcal/kg) (d)	29.0 (20.3, 36.0)	23.0 (16.0, 33.0)	–4.662	0.000	28.0 (20.0, 39.0)	19.0 (13.0, 28.0)	–14.824	0.000
**Amino acid M (P25, P75)**								
Start time (d)	1.0 (1.0, 2.0)	1.0 (1.0, 1.0)	–0.628	0.530	1.0 (1.0, 2.0)	1.0 (1.0, 1.0)	–2.028	0.053
Age at reaching 3.0–3.5 g/kg/day (d)	5.0 (4.0, 7.0)	5.0 (4.0, 6.0)	–2.124	0.034	5.0 (4.0, 7.0)	5.0 (4.0, 6.0)	–6.602	0.000
Starting dose (g/kg)	1.5 (1.0, 2.0)	1.5 (1.0, 1.8)	–1.082	0.279	1.5 (1.0, 1.9)	1.5 (1.0, 1.8)	–0.244	0.807
Cumulative dose during W1 (g/kg)	17.2 (14.9, 19.6)	16.0 (13.3, 18.4)	–4.198	0.000	16.2 (13.6, 18.5)	16.0 (12.9, 18.2)	–3.219	0.001
Cumulative dose during hospitalization (g/kg)	67.0 (40.3, 89.4)	44.1 (25.5, 71.0)	–6.250	0.000	51.6 (33.5, 82.3)	38.0 (21.2, 59.0)	–11.742	0.001
**Fat emulsions M (P25, P75)**								
Start time (d)	2.0 (1.0, 2.0)	2.0 (1.0, 2.0)	–1.263	0.207	2.0 (1.0, 2.0)	2.0 (1.0, 2.0)	–0.871	0.384
Age at reaching 3.0 g/kg/day (d)	5.0 (4.0, 7.0)	5.0 (4.0, 7.0)	–1.245	0.213	5.0 (4.0, 7.0)	5.0 (4.0, 6.0)	–4.031	0.000
Starting dose (g/kg)	1.0 (1.0, 1.1)	1.0 (1.0, 1.0)	–0.750	0.453	1.0 (1.0, 1.1)	1.0 (1.0, 1.0)	–1.254	0.210
Cumulative dose during W1 (g/kg)	13.3 (10.7, 15.2)	12.5 (10.0, 15.0)	–2.196	0.028	13.0 (10.5, 15.0)	12.2 (9.5, 14.8)	–5.536	0.000
Cumulative dose during hospitalization (g/kg)	47.1 (33.2, 74.6)	37.0 (20.6, 59.5)	–4.8432	0.000	44.5 (28.0, 71.6)	32.0 (16.7, 51.1)	–11.594	0.000
**HMF M (P25, P75)**								
Breast milk volume on addition of HMF (ml/kg)	100.0 (88.7, 121.9)	102.9 (90.0, 124.4)	–0.453	0.650	108.0 (94.9, 130.0)	100 (88.0, 120.0)	–4.853	0.000
Days for full fortification (d)	10.0 (6.0, 20.0)	8.0 (4.0, 18.0)	–1.573	0.116	11.0 (5.0, 20.0)	6.0 (4.0, 16.0)	–6.868	0.000
**Other nutrients**								
Microelements [*n* (%)]	100 (75.2)	1,858 (78.2)	0.651	0.420	846 (80.0)	1,012 (76.7)	3.605	0.058
Calcium [n (%)]	88 (66.2)	1,389 (58.4)	3.147	0.076	652 (61.7)	737 (55.7)	8.516	0.004
Phosphorus [*n* (%)]	92 (69.2)	1,573 (66.1)	0.535	0.465	695 (65.7)	878 (66.4)	0.138	0.711

SGA, small for gestational age; EUGR, extrauterine growth retardation; true EUGR, infants in the EUGR and non-SGA group; h, hour; d, day; EN, enteral nutrition; PN, parenteral nutrition; W1, the first week after birth; HMF, human milk fortifier.

### Univariate analysis of main therapies and complications of “true extrauterine growth retardation” evaluated by weight

The results of univariate analysis of complications during hospitalization showed that there were statistically significant differences in the incidences of RDS, BPD, moderate to severe BPD, postnatal corticosteroid use, FI, PNAC, MBDP, anemia, and transfusion between the groups (SGA vs. non-SGA group, and EUGR vs. non-EUGR group) (all *P* < 0.05). Nosocomial infection, EOS, brain injury, IVH, and PVL were not significant. With respect to LOS, NEC ≥ stage 2, PDA, hsPDA, ROP, and ROP requiring intervention, the differences were not significant between the SGA and non-SGA groups at birth, but they became significant between the EUGR and non-EUGR groups belonging to the non-SGA group at discharge (*P* < 0.05). The results are presented in Table 4.

**TABLE 4:** Comparisons of therapies and complications between the groups.

	At birth (*n* = 2,514)		X	*P*	Non-SGA group (*n* = 2,381)		X	*P*
	SGA (*n* = 133)	Non-SGA (*n* = 2,381)			EUGR (true EUGR) (*n* = 1,058)	Non-EUGR (*n* = 1,323)		
**Main therapies M (P25, P75)**								
Invasive MV time (d)	0.2 (0.0, 4.0)	0.5 (0.0, 4.0)	–0.368	0.713	2.0 (0.0, 6.0)	0.0 (0.0, 2.0)	–11.686	0.000
Non-invasive MV time (d)	19.0 (8.0, 31.5)	15.0 (6.0, 28.0)	–2.237	0.025	19.0 (8.0, 31.0)	11.0 (5.0, 24.0)	–9.737	0.000
Oxygen time (d)	11.0 (4.0, 23.0)	8.0 (2.0, 17.5)	–2.684	0.007	10.0 (3.0, 19.0)	7.0 (1.0, 16.0)	–5.293	0.000
Cumulative time of antibiotics (d)	14.0 (7.0, 22.0)	12.0 (7.0, 20.0)	–1.305	0.192	14.0 (7.0, 22.0)	10.0 (7.0, 17.0)	–8.013	0.000
**Main comorbidities**								
RDS [*n* (%)]	105 (78.9)	1,636 (68.7)	6.167	0.013	761 (72.0)	875 (66.1)	9.385	0.002
BPD [*n* (%)]	78 (58.6)	1,074 (45.1)	9.302	0.002	566 (53.5)	508 (38.4)	54.132	0.000
Moderate to severe BPD [*n* (%)]	30 (22.6)	377 (15.8)	4.196	0.041	235 (22.2)	142 (10.7)	58.122	0.000
Postnatal corticosteroids [*n* (%)]	31 (23.3)	319 (13.4)	10.368	0.006	190 (18.0)	129 (9.8)	35.492	0.000
EOS [*n* (%)]	20 (15.0)	349 (14.7)	0.015	0.904	163 (15.4)	186 (14.1)	0.853	0.356
LOS [*n* (%)]	18 (13.5)	309 (13.0)	0.029	0.864	183 (17.3)	126 (9.6)	31.364	0.000
Nosocomial infection [*n* (%)]	26 (19.5)	392 (16.5)	0.865	0.352	178 (16.8)	214 (16.2)	0.180	0.671
NEC ≥ stage 2 [*n* (%)]	14 (10.5)	198 (8.3)	0.797	0.372	123 (11.6)	75 (5.7)	27.360	0.000
Brain injury [*n* (%)]	41 (30.8)	948 (39.8)	4.264	0.050	414 (39.1)	534 (40.4)	0.373	0.542
IVH [*n* (%)]	38 (28.6)	819 (34.4)	1.903	0.168	353 (33.4)	466 (35.2)	0.899	0.343
IVH III-IV [*n* (%)]	2 (1.5)	49 (2.1)	0.195	0.659	24 (2.3)	25 (1.9)	0.418	0.518
PVL [*n* (%)]	3 (2.3)	94 (3.9)	0.972	0.324	49 (4.6)	45 (3.4)	2.345	0.126
PDA [*n* (%)]	69 (51.9)	1214 (51.0)	0.040	0.841	570 (53.9)	644 (48.7)	6.356	0.012
hsPDA [*n* (%)]	36 (27.1)	745 (31.3)	1.048	0.306	378 (35.7)	367 (27.7)	17.447	0.000
ROP [*n* (%)]	49 (36.8)	727 (30.5)	2.339	0.126	385 (36.4)	342 (25.9)	30.653	0.000
ROP requiring intervention [*n* (%)]	6 (4.5)	74 (3.1)	0.803	0.370	46 (4.3)	28 (2.1)	9.699	0.002
FI [*n* (%)]	65 (48.9)	850 (35.7)	9.442	0.002	458 (43.3)	392 (29.6)	47.784	0.000
MBDP [*n* (%)]	8 (6.0)	64 (2.7)	5.012	0.025	36 (3.4)	28 (2.1)	3.718	0.044
PNAC [*n* (%)]	23 (17.3)	239 (10.0)	7.103	0.008	152 (14.4)	87 (6.6)	39.514	0.000
Anemia [*n* (%)]	124 (93.2)	1,992 (83.7)	8.659	0.003	955 (90.3)	1,037 (78.4)	60.724	0.000
Transfusion [*n* (%)]	104 (78.2)	1,398 (58.8)	19.735	0.000	771 (73.1)	627 (47.4)	159.868	0.000

SGA, small for gestational age; EUGR, extrauterine growth retardation true EUGR, infants in the EUGR and non-SGA group; d, day; MV, mechanical ventilation; RDS, respiratory distress syndrome; BPD, bronchopulmonary dysplasia; EOS, early-onset sepsis; LOS, late-onset sepsis; NEC, necrotizing enterocolitis; IVH, intraventricular hemorrhage; PVL, periventricular leukomalacia; PDA, patent ductus arteriosus; hsPDA, hemodynamically significant patent ductus arteriosus; ROP, retinopathy of prematurity; FI, feeding intolerance; MBDP, metabolic bone disease of prematurity; PNAC, parenteral nutrition associated cholestasis.

### Multivariate logistic regression analysis of the risk factors for “true extrauterine growth retardation”

In this multicenter study, multivariate analysis showed that the cumulative caloric intake during the first week and GV were protective factors for EUGR, while long duration of fasting, days to reach total EN, late initiation of HMF, and full fortification were independent risk factors for EUGR. In terms of perinatal factors and postnatal complications, multivariate analysis showed that SGA and moderate to severe BPD were independent risk factors for EUGR. In non-SGA infants, cumulative caloric intake during the first week was a protective factor for “true EUGR”, while days to reach total EN, late initiation of HMF, and moderate to severe BPD were independent risk factors for “true EUGR”. The results are presented in Table 5.

**TABLE 5:** Multivariate logistic regression analysis of the risk factors for the occurrence of “true EUGR.”

Variable	*B*	SE	Wald X^2^	*P*	OR (95% CI)
Birth weight	0.101	0.027	14.382	0.000	1.150 (1.018–1.295)
Days to reach total EN	0.657	0.433	6.324	0.005	2.335 (1.212–3.546)
Cumulative calorie intake during W1	–0.015	0.020	6.210	0.012	0.920 (0.913–0.945)
The volume of breast milk on addition of HMF was added	0.135	0.208	40.355	0.000	2.122 (1.523–2.836)
Moderate to severe BPD	0.768	0.375	7.281	0.000	3.423 (1.041–5.645)

EUGR, extrauterine growth retardation; true EUGR, infants in the EUGR and non-SGA group; EN, enteral nutrition; W1, the first week after birth; HMF, human milk fortifier; BPD, bronchopulmonary dysplasia; OR, odds ratio, CI, confidence interval.

## Discussion

Very preterm infants account for 10% of all preterm births (1). Recent studies in China have shown that VPI account for 13.1% of all preterm infants and EPI account for 1.1% of all preterm infants. The success rate of treatment of VPI in China is 93.3% (15), and the quality of life after treatment has attracted increasing attention (16, 17). Thus, it is very important to analyze the risk factors for “true EUGR” in China and optimize the comprehensive hospital management strategy to improve the short-term and long-term prognoses of VPI.

In this study, the incidence of EUGR evaluated by weight, length, and HC at discharge in the SGA and non-SGA groups was statistically different. The incidences of “true EUGR” and severe “true EUGR” evaluated by weight were 43.5 and 18.9% at discharge, respectively. A national multicenter study performed in 2009 in China showed that among 696 singleton preterm infants, the incidence of EUGR in the non-SGA group was 47.4% (18). Another provincial multicenter study performed in 2020 showed that the incidence of “true EUGR” was 52.9% among 1,051 very low birth weight infants (VLBWIs) (19). Lima et al. (20) studied 570 preterm infants weighing < 1,500 g, and they found that, at neonatal intensive care unit discharge, the incidence of EUGR was 12.3% in non-SGA infants. Figueras-Aloy et al. (2) showed that among 479 VPI, EUGR occurred in 50.7% at 34–36 postmenstrual weeks, but the percentage decreased to 21.1% at 2–2.5 years; however, if SGA infants were excluded from the group (“true-EUGR”), the corresponding values would be 42.7 and 15.4%, respectively.

In this study, the variables independently related to the presence of “true EUGR” were low BW, days to reach total EN, late initiation of HMF, and moderate to severe BPD. Lima et al. (20) highlighted the importance of SGA, RDS, and severe BPD. Low BW with greater GA, RDS, BPD, and male sex as EUGR factors were described by Figueras-Aloy et al. (2). Our findings confirmed the fact that BW is a risk factor for EUGR. It is reasonable to infer that organ function will develop better and the possibility of FI and underlying diseases will decrease as infants mature (10). In preterm infants with a lower BW, the accumulation of intrauterine nutrients is less and complications related to preterm birth are more likely to occur after birth, resulting in higher nutritional requirements and higher energy metabolism consumption, which are more likely to lead to nutritional deficiency, thus causing EUGR (21).

Increasing the energy intake in the first week of postnatal life is associated with postnatal weight gain and reduced risk of EUGR and BPD (22, 23). The updated 2013 version of the Chinese guidelines for nutrition support in neonates has recommended a daily calorie intake of 120 kcal/kg/day. Specifically, the recommended total calorie intake is 105–130 kcal/kg/day for neonates, 110–135 kcal/kg/day for preterm infants, and up to 150 kcal/kg/day for VLBWIs. For every 10 kcal/kg/day increase in energy intake on the 4–6th day of life (DOL), the weight standard deviation score on DOL7 increased by 0.08, and the risk of EUGR decreased correspondingly (OR 0.73, 95% CI 0.66–0.82) (22, 23). A cohort study of VPI in Spain (24) showed that the EUGR group had a lower energy intake in the first week of life and slower GV than the non-EUGR group, (8.6 ± 4.0 vs. 13.8 ± 5.0 g/kg/day), which is consistent with the conclusion of this study (14.2 vs. 15.9 g/kg/day). Ventilator dependence on DOL7, early and persistent pulmonary dysfunction, and dexamethasone exposure were associated with low GV at 2–4 weeks (25).

Early attainment and maintenance of adequate EN can promote growth and weight recovery in preterm infants, and it can reduce the short-term and long-term complications (8, 26). A long time required to reach total EN is an independent risk factor for “true EUGR” in VPI. Further, inadequate EN can cause gastrointestinal mucosal atrophy, increase the permeability and decrease the repair ability, increase the incidence of stress-induced gastrointestinal bleeding and FI, inadequate protein and energy intake, aggravate the cumulative nutrient loss, and increase the risk of EUGR at discharge. Parenteral nutrition was found to be a negative predictor of insulin-like growth factor-1 levels, and there could potentially be a time frame in which the macronutrient intake does not affect the insulin-like growth factor-1 levels, which plays an important role in the complex association between nutrition, growth, and maturation in EPI and VPI (27).

In this study, multivariate logistic regression analysis showed that late initiation of HMF was an independent risk factor for “true EUGR.” Early addition of HMF can increase protein intake within 4 weeks after birth and reduce early protein accumulation deficiency, and it does not increase the chances of complications, such as NEC or FI (28, 29). Foreign research on HMF has mainly focused on the VLBWI population. Based on the current situation of insufficient EN support and high incidence of EUGR in preterm infants in China, Expert Consensus on the Use of HMF for Preterm Infants (30) has expanded the application of HMF to preterm infants with BW < 1,800 g. The consensus has suggested that HMF should be introduced when the breastfeeding amount reaches 50–80 ml/kg/day, and full fortification should be achieved within 3–5 days if tolerated. Although there is consensus on the addition of HMF in breast milk, in this study, HMF was added at a later time point in both the EUGR group and the non-EUGR group (108 vs. 100 ml/kg) and the time to reach full enhancement of HMF was longer (11 vs. 6 days); the difference between the two groups was statistically significant. This suggests that there are still many irregularities in the use of HMF in China. Therefore, the relevant nutrition guidelines for preterm infants should be followed in clinical practice. Under the premise of FI, the milk volume should be increased as soon as possible, HMF should be added in the early stage, and full fortification should be achieved as soon as possible.

Multivariate logistic regression analysis showed that moderate to severe BPD was an independent risk factor for “true EUGR.” Several studies have supported the claim that moderate to severe BPD was associated with more severe EUGR (31, 32). Long-term respiratory support and increased work of breathing, chronic stress, liquid limit, and diuretics and glucocorticoid use in infants with BPD lead to inadequate energy intake. The body is in a highly decomposed state, with increased energy consumption, and there is insufficient intrauterine storage, which leads to a negative nitrogen balance, prolonged time to reach total EN, and slower weight gain; thus, leading to a higher incidence of EUGR (22, 31).

Domestic and foreign guidelines have been revised and updated in the last 10 years, with the management of EN and PN becoming more active and standardized. Most of the previous multicenter studies have focused on VLBWI (9, 33), and this is the first study to focus on the incidence and risk factors of “true EUGR” in VPI in China. The study design was of a prospective multicenter study, and the data on perinatal information, weight, length, HC, and other growth indicators of VPI were relatively complete and reliable. Although several studies have reported EUGR in larger samples, they were limited by the retrospective nature of the studies. The results of this study can provide a basis for better understanding and improving the nutritional status of VPI in China and optimizing the nutritional support program. The limitations of this study include the vast territory of China and data from seven different regions and 28 hospitals, which may have led to variations in the nutrition management strategies of each hospital.

## Conclusion

Over the past 10 years, enteral nutrition and parenteral nutrition of VPI have made great progress, and the incidence of “true EUGR” has decreased significantly. However, it should be emphasized that perinatal care should continue to be strengthened to further reduce the occurrence of SGA. Cumulative caloric intake should be ensured and increased during the first week of hospitalization. More attention should be paid toward days to reach total enteral nutrition, initiation of HMF, and moderate to severe BPD to reduce the risk of “true EUGR.”

## Data availability statement

The raw data supporting the conclusions of this article will be made available by the authors, without undue reservation.

## Ethics statement

The studies involving human participants were reviewed and approved by Women and Children’s Hospital, School of Medicine, Xiamen University (KY-2019-016). Written informed consent to participate in this study was provided by the participants’ legal guardian/next of kin.

## Author contributions

X-ZL, X-MT, and CC: conceptualization, writing - review and editing, and project administration. WS, Y-MC, RZ, and ZZ: methodology and data curation. WS, X-ZY, and Y-PQ: software. LM, RC, HW, and D-MC: validation. WS and FW: formal analysis. FW, JM, LL, Y-MC, RZ, ZZ, X-ZY, Y-PQ, LM, RC, HW, and D-MC: investigation and resources. WS: writing—original draft preparation. FW, JM, and LL: visualization and supervision. All authors have read and agreed to the published version of the manuscript.
